# Exosomes regulate SIRT3-related autophagy by delivering miR-421 to regulate macrophage polarization and participate in OSA-related NAFLD

**DOI:** 10.1186/s12967-024-05283-8

**Published:** 2024-05-19

**Authors:** Li Yang, Shijie Liu, Yan He, Lulu Gan, Qing Ni, Anni Dai, Changhuan Mu, Qian Liu, Hongyan Chen, Hongying Lu, Ruixue Sun

**Affiliations:** 1 Hypertension Center, Yan ‘an Hospital of Kunming Medical University, 245 Renmin East Road, Panlong District, Kunming City, 650000 Yunnan Province China; 2 Kunming Technical Diagnosis and Treatment Center for Refractory Hypertension, Kunming Medical University, 245 Renmin East Road, Panlong District, Kunming City, 650000 Yunnan Province China

**Keywords:** Obstructive sleep apnea, Non-alcoholic fatty liver, Exosomes, Macrophages, miR-421, SIRT3/AMPK, Autophagy

## Abstract

**Purpose:**

To analyze the role of and mechanism underlying obstructive sleep apnea (OSA)-derived exosomes in inducing non-alcoholic fatty liver (NAFLD).

**Methods:**

The role of OSA-derived exosomes was analyzed in inducing hepatocyte fat accumulation in mice models both in vivo and in vitro.

**Results:**

OSA-derived exosomes caused fat accumulation and macrophage activation in the liver tissue. These exosomes promoted fat accumulation; steatosis was more noticeable in the presence of macrophages. Macrophages could internalize OSA-derived exosomes, which promoted macrophage polarization to the M1 type. Moreover, it inhibited sirtuin-3 (SIRT3)/AMP-activated protein kinase (AMPK) and autophagy and promoted the activation of nucleotide-binding domain, leucine-rich–containing family, pyrin domain–containing-3 (NLRP3) inflammasomes. The use of 3-methyladenine (3-MA) to inhibit autophagy blocked NLRP3 inflammasome activation and inhibited the M1 polarization of macrophages. miR-421 targeting inhibited SIRT3 protein expression in the macrophages. miR-421 was significantly increased in OSA-derived exosomes. Additionally, miR-421 levels were increased in OSA + NAFLD mice- and patient-derived exosomes. In the liver tissues of OSA and OSA + NAFLD mice, miR-421 displayed similar co-localization with the macrophages. Intermittent hypoxia-induced hepatocytes deliver miR-421 to the macrophages via exosomes to inhibit SIRT3, thereby participating in macrophage M1 polarization. After OSA and NAFLD modeling in miR-421^−/−^ mice, liver steatosis and M1 polarization were significantly reduced. Additionally, in the case of miR-421 knockout, the inhibitory effects of OSA-derived exosomes on SIRT3 and autophagy were significantly alleviated. Furthermore, their effects on liver steatosis and macrophage M1 polarization were significantly reduced.

**Conclusions:**

OSA promotes the delivery of miR-421 from the hepatocytes to macrophages. Additionally, it promotes M1 polarization by regulating the SIRT3/AMPK-autophagy pathway, thereby causing NAFLD.

**Supplementary Information:**

The online version contains supplementary material available at 10.1186/s12967-024-05283-8.

## Introduction

Obstructive sleep apnea (OSA) is a severe sleep-breathing disorder characterized by intermittent hypoxia during sleep [1]. Clinical trials have confirmed that OSA can cause insulin resistance, obesity, and metabolic syndrome [2–4]. Of them, non-alcoholic fatty liver (NAFLD) is the primary complication of OSA [5]. OSA has been identified as a high-risk factor for NAFLD [6–8]. However, the mechanism by which OSA causes NAFLD is unclear.

Exosomes are important paracrine regulatory elements [9]. These membrane vesicles are secreted by most cells; their diameter ranges from 30 nm to 200 nm [9, 10]. The lipid bilayer membrane of exosomes can protect the contents of proteins, mRNA, and microRNA (miRNA) to be delivered between the cells. Furthermore, they can easily penetrate the blood-brain barrier [11, 12]. The miRNA level in the plasma exosomes of patients with OSA changes significantly and affects fat cell metabolism [13]. In children with OSA and endothelial dysfunction, miR-630 is downregulated in the peripheral blood exosomes. Moreover, it is involved in obesity and vascular dysfunction [14]. Therefore, OSA-derived exosomes can be delivered to the entire body and participate in OSA-related complications.

Macrophages are central to NAFLD. Liver-resident Kupffer cells (KC) and recruited macrophages get activated and transformed to the M1 phenotype to exert a pro-inflammatory response. Autophagy can decompose the nucleotide-binding domain, leucine-rich–containing family, and pyrin domain–containing-3 (NLRP3) inflammasome to alter the balance of M1/M2 to M2, thereby alleviating NALFD [15]. Sirtuin-3 (SIRT3)/AMP-activated protein kinase (AMPK) can inhibit the NLRP3 inflammasomes by promoting autophagy, thereby impeding macrophage polarization to M1 [16, 17]. However, the role of the SIRT3/AMPK-autophagy-NLRP3 pathway in OSA-induced NAFLD and its regulatory mechanism are unclear.

Interestingly, macrophages receive exosomes and are regulated by them. Alcohol stimulates macrophage activation by promoting the release of CD40 ligand-rich exosomes from the hepatocytes [18]. Cholesterol promotes the release of exosomes comprising abundant miR-122-5p from the hepatocytes, leading to the polarization of macrophage M1 [19]. Lipotoxic hepatocytes deliver miR-192-5p to the macrophages through exosomes, thereby regulating the protein kinase B (AKT)/forkhead box O (FOXO1) pathway. This phenomenon activates the macrophages and promotes NAFLD [20]. However, the role of hepatocytes in regulating OSA-derived exosomes remains unclear.

In OSA, hepatocytes are affected by hypoxia. Moreover, hypoxia induces the release of exosomes and abnormal contents [16]. Therefore, chemical hypoxia can induce hepatocytes in OSA [15]. Thus, we aimed to analyze whether the exosomes secreted by hepatocytes in OSA affect macrophage polarization. Moreover, we intended to explore the molecular mechanism by which the miRNAs in exosomes affect macrophage polarization.

## Materials and methods

### Blood sample

The study participants included patients with OSA, patients with OSA combined with NAFLD, and healthy individuals (*n* = 20 each). The inclusion criteria were as follows: (1) age between 20 years and 60 years; (2) diagnosed with OSA or NAFLD; and (3) first diagnosed disease. The exclusion criteria were as follows: (1) liver diseases; (2) respiratory diseases; and (3) infections, malignant tumors, hematological diseases, hypertension, diabetes, and other systemic diseases. Briefly, 2 mL of peripheral blood was collected, rapidly transferred into a tube of ethylenediamine tetraacetic acid, and mixed by vortexing or using a pipette tip. Within 1 h, the samples were centrifuged at 8,200 × *g* (9,400 rpm) for 10 min at 4 °C. One milliliter of the supernatant was pipetted and transferred to a clean 1.5 mL centrifuge tube. It was centrifuged at 16,000 × *g* (13,200 rpm) for 10 min at 4 °C. The supernatant was carefully pipetted into a new centrifuge tube. The samples were stored in a -80 °C refrigerator. All participants provided their informed consent for participation before enrollment. The study was approved by the Ethics Committee of Yan’an Hospital Affiliated with the Kunming Medical University.

### Animal grouping

Ninety 4-week-old C57BL/6J male mice (SPF, Shanghai Slack Experimental Animal Center, China) and miR-421^−/−^ mice (Linmei Biological Technology Co., Ltd. Hefei, China) were raised in a pathogen-free environment, alternating light and dark cycle for 12 h (8:00 am–8:00 pm). The NAFLD model was constructed by feeding the mice a high-fat diet (32.1% carbohydrates, 16.5% protein, and 51.4% fat) for 6 weeks. A normal diet consisted of 32.1%, 16.5%, and 51.4% carbohydrates, protein, and fat, respectively. This experiment was conducted per the Ethics Committee of Yan’an Hospital Affiliated with the Kunming Medical University. The animal experiment guidelines and research plan were approved by the Animal Experiment Committee of Yan’an Hospital Affiliated with the Kunming Medical University.

In Group 1 (*n* = 6), the mice were divided into Control, Control-50, Control-100, OSA-50, and OSA-100 types. After 1 week of adaptation, healthy mice were intraperitoneally injected with exosomes from healthy or OSA mice (50–100 µg/mouse). The injection was administered once every 3 days. Six weeks later, peripheral blood was collected; liver tissues were collected after euthanasia.

In Group 2 (*n* = 6), the mice were divided into Healthy-Control, Healthy-OSA, Healthy-OSA + NAFLD, miR-421^−/−^-Control, miR-421^−/−^-OSA, and miR-421^−/−^-OSA + NAFLD types. Healthy and miR-421 gene knockout mice were used to construct the OSA and OSA + NAFLD models, respectively. Liver tissues were collected 6 weeks after euthanasia.

In Group 3 (*n* = 6), the mice were divided into Control, OSA, and OSA + miR-421^−/−^ types. The exosomes from healthy, OSA, and miR-421^−/−^ OSA mice were used to treat the healthy mice (100 µg/mouse). The injection was administered once every 3 days. Liver tissues were collected 6 weeks after euthanasia.

### Exosome collection and labeling

ExoQuick and ExoQuick-TC Kits (System Biosciences, Mountain View, CA) were used for exosome collection. First, 500 µL of fresh human/mouse serum or 10 mL of cell culture medium was mixed with the reagent and separated according to the manufacturer’s instructions. The exosomes were labeled using PKH26 (Sigma-Aldrich, St. Louis, USA). Second, these exosomes (50 µL) and PKH26 (1 µL) were added to Reagent C and incubated for 5 min at 25 ℃. Third, 1% bovine serum albumin (BSA) (150 µL) was added to neutralize the excess PKH26. The exosomes were purified using an exosomal spin column (Invitrogen, Carlsbad, CA, USA). The morphology of separated exosomes was observed under an electron microscope. The particle size distribution of the exosomes was evaluated using a particle size analyzer (NanoSight LM10, Malvern, UK). Western blotting was conducted to detect the expression of exosomal marker proteins CD9, CD63, and CD81, and exosome-negative marker protein Calnexin.

### OSA model

In this study, chronic intermittent hypoxia (CIH) was used to construct an OSA model. The induction principle of OSA-related diseases, including metabolic dysfunction-associated steatotic liver disease, was based on OSA-induced chronic hypoxia [21–23]. OSA could decrease O_2_ inhalation directly. Therefore, OSA-induced hypoxia was simulated by directly controlling the inhaled O_2_ concentration, i.e., CIH was used to simulate OSA. The inhaled O_2_ concentration was controlled by placing the mice in a glass chamber (30 × 20 × 20 in, Oxycycler model A44XO, BioSpherix, Redfield, NY, USA). After 1 week of acclimatization, they received daily intermittent hypoxia treatment for 8 h. The chamber outlet was connected to an instrument detecting O_2_ concentration. Based on the outlet concentration, the online control system adjusted the air and nitrogen flow at the inlet, such that the average O_2_ concentration was 10 ± 1% during hypoxia. In the absence of light, the O_2_ concentration was maintained at 10% for 2 min. Subsequently, it was restored to normoxic conditions (21%) alternately for 8 h. The remaining time involved normoxia conditions. Intermittent hypoxia lasted for 6 weeks.

### Hematoxylin and eosin staining

The liver specimens were fixed in 10% neutral formalin, sectioned, and deparaffinized. After adding hematoxylin, the specimens were incubated at 25 ℃ for 10 min. Subsequently, 0.5% eosin solution was added; the specimens were incubated at the same temperature for 3 min. Lesions of the colon cross-section were observed under a microscope (Olympus, Japan).

### RNA fluorescence in situ hybridization

miR-421 localization in the liver tissues was detected using fluorescence in situ hybridization. First, the paraffin sections were deparaffinized, and a freshly diluted pepsin stock solution containing 3% citric acid was added. The sections were digested at 37 °C for 1 min. Second, 4% paraformaldehyde containing 0.1% diethyl pyrocarbonate (pH = 7.2–7.6) was added to the sections. Moreover, 20 µl of pre-hybridization solution was added; the sections were incubated for 2 h. Third, a fluorescein-labeled (red fluorescent) miR-421 probe (Shenggong Biological Engineering Co., Ltd., Shanghai, China) was added and incubated overnight at 37 °C. Finally, 4′,6-diamidino-2-phenylindole (DAPI) was added for nucleus staining. The sections were observed under a laser confocal microscope.

### Isolating primary hepatocytes and KCs

The primary hepatocytes and macrophages were isolated [24, 25]. A collagenase/protease mixture (Vitacyte, Indianapolis, IN) was applied for the in-situ perfusion of mouse liver to enable digestion. Liver tissues were placed in a 150-mm petri dish and operated in a sterile environment. The petri dish contained 1% BSA, 15 mM Hanks’ Balanced Salt Solution (HBSS), and 1 g/L glucose (Gibco, Carlsbad, CA, USA). The liver parenchymal cells were shed by gentle shaking. The sample was passed through a filter (105 μm) and centrifuged (4 °C, 70 × *g*, 3 min). The pellet was used to separate the hepatocytes. Subsequently, the supernatant was used to separate the macrophages. The hepatocytes (purity > 99%) were collected using Percoll density gradient centrifugation (GE Healthcare, Marlborough, MA).

The supernatant and sample containing nonparenchymal cells were passed through the filter (297 μm) and centrifuged (4 °C, 500 × *g*, 6 min). The particles containing nonparenchymal cells were resuspended in HBSS and passed through a 70 μm cap filter. Furthermore, Avanti J-26XP centrifuge (Beckman-Coulter, Brea, CA), Optiprep density gradient centrifugation (Sigma-Aldrich), and countercurrent elutriation centrifugation were used to separate the macrophages from other nonparenchymal cells. The purity of macrophages was determined using flow cytometry (> 95%).

### Hepatocyte culture and treatment

The hepatocytes were cultured in Dulbecco’s Modified Eagle Medium/F12 medium (Gibco, Carlsbad, CA, USA) with 10% fetal bovine serum (Gibco, Carlsbad, CA, USA) in a 5% CO_2_ incubator at 37 °C.

To induce NAFLD in vitro, a free fatty acid (FFA) mixture (oleate: palmitate = 2:1) (0.5 mmol/L concentration) was added to the culture medium and incubated for 24 h. Additionally, 20 µg/mL of exosomes were added to the medium to induce the hepatocytes.

To induce intermitted hypoxia in vitro [26], the O_2_ concentration in the incubator was adjusted by modifying the N_2_ content. Moreover, CO_2_ concentration was maintained at 5%. Every 30 min, the O_2_ concentration was changed from 1 to 21%. The cells were incubated for 6 days.

### Macrophage culture and treatment

The PKH26-labeled exosomes were red fluorescent. Briefly, 20 µg/mL of exosomes were added to the culture medium to induce the macrophages. After 24 h of induction, the macrophages were fixed and DAPI (Sigma) was added for nucleus counterstaining. The internalization of macrophages in exosomes was observed under a confocal microscope (Zeiss LSM 700, Germany).

Lipopolysaccharides (LPS) were used to activate the NLRP3 inflammasomes and M1 polarization. Approximately 1 mg/mL of LPS (L2630, Sigma-Aldrich) was added to the medium and incubated for 4 h. The proteasome inhibitor MG-132 (S2619, Selleck, USA) was added to the medium to inhibit degradation. Subsequently, 50 µmol/L of 3-methyladenine (3-MA, S2767, Selleck) was added to inhibit autophagy.

### Cell co-culture

Forty-eight-well type I collagen-coated plates were used to construct a co-culture system of hepatocytes and macrophages. Hepatocytes and macrophages were used in a ratio of 2:1 [27]. First, hepatocytes were added at a density of 4 × 10^5^ cells/well and shaken gently every 15 min. Second, macrophages were added at a density of 2 × 10^5^/well and shaken gently every 15 min. After four times, the medium was removed; fresh medium was added and cultured.

### Cell transfection

The miR-421 mimic and corresponding negative control (NC) plasmids were obtained from GenePharma (Shanghai, China). The cells were transfected with 50 nM mimic and inhibitor using Lipofectamine™ 2000 (Invitrogen, Waltham, USA).

### Dual luciferase report

The 3’-UTR sequence of wild-type (wt-) SIRT3 mRNA was amplified to the downstream site of the pGL4 luciferase vector (Promega, Madison, WI, USA). To generate the mutated (mut-) SIRT3 mRNA 3’-UTR, the rapid site-directed mutagenesis kit (D0206, Beyotime) was used. The macrophages were seeded in 24-well plates at a density of 3 × 10^4^/well. After 24 h, 1 µg of wt- or mut-SIRT3 luciferase plasmid, 50 nM of miR-421 mimic or miR-421 NC, and 150 ng of Renilla luciferase plasmid (Beyotime) were transfected into the macrophages via Lipofectamine^TM^2000. The cells were incubated at 37 °C for 36 h. Following the manufacturer’s protocol, a dual luciferase reporter gene detection kit (Promega, Madison, WI, USA) was used to detect luciferase activity. All data were normalized to detect Renilla luciferase activity.

### Triglyceride and cholesterol measurement

The levels of triglyceride (TG) and total cholesterol (TC) in the liver tissues and cells were detected using an enzymatic assay kit (E1025-105, E1026-105, Applygen Technologies Inc., Beijing, China).

### Oil red O staining

First, Oil red O (ORO) reagent (C0158M, Beyotime, Shanghai, China) was added to the liver Sect. (10 μm) and stained at 25 ℃ for 20 min. Second, the cells were stained using hematoxylin (C0107M, Beyotime) and incubated for 5 min for nucleus staining. The cells were treated using 10% formalin for fixation (15 min). They were covered using a washing solution and incubated for 20 min. After rinsing the washing solution, ORO reagent was added. The cells were incubated at 25 ℃ for 20 min. After washing with phosphate-buffered saline (PBS) for 20 s, hematoxylin was added for counterstaining.

### Transmission electron microscopy

Autophagosomes in the cells were observed via transmission electron microscopy (TEM). The cells were fixed with 2.5% glutaraldehyde solution overnight at 4 °C and 1% osmic acid for 2 h. After washing thrice with PBS, the cells were embedded in Epon/Araldite, 1.5% uranyl acetate, and 2% lead citrate. Autophagosomes were observed and pictures were captured using TEM (JEM 1010, JEOL, MA, USA).

### Immunofluorescence staining

First, the tissues and cell slides were fixed in paraformaldehyde overnight, dehydrated, and frozen with an embedding agent on a -20 °C freezing table. The frozen tissue blocks were smoothed and cut into 4-µm-thick slices. Second, the temperature was changed to 25 ℃. The samples were washed thrice with 1X PBS for 5 min each. The samples were soaked completely in 0.01 M citrate buffer (pH = 6.0), heated in a microwave oven over medium-low heat for repair, and maintained in a moderate boiling state for 5 min for the best effect. Third, permeabilization was conducted using 0.5% Triton X-100 (prepared in 1 X PBS) for 20 min at 25 ℃. F4/80 (ab111101, 1: 100), CD86 (ab119857, 1:100), and CD206 (ab300622, 1:100) were added to the samples. They were incubated overnight at 4 °C. Phycoerythrin/ fluorescein isothiocyanate-conjugated secondary antibody (Abcam) was added to the samples and incubated for 1 h. Finally, DAPI (Sigma) was added for nucleus counterstaining. Antifade-containing glycerol was used for mounting. Protein localization in the cells was observed under a laser confocal microscope (FV 1200, Faxitron, US).

### Quantitative reverse transcription polymerase chain reaction

Total RNAs from the tissues and cells were acquired using TRIzol (Sigma, St. Louis, MO, USA), and the purities were detected. For mRNA, reverse transcription (60 min at 42 °C and 5 min at 70 °C; stored at 4 °C) and quantitative polymerase chain reaction (40 cycles at 95 °C for 10 min, 94 °C for 15 s, 60 °C for 1 min, and 60 °C for 1 min; stored at 4 °C) were performed using the PrimeScript-RT kit (Takara, Japan) and SYBR Premix Ex Taq™ kit (Takara, Japan), respectively. Glyceraldehyde 3-phosphate dehydrogenase (GAPDH) was used as the standardized reference. 2^*−ΔΔCt*^ was used to analyze the relative expression of the target RNAs [28].

For miR-421, the total miRNA was extracted using the miRNeasy Mini kit (GE Healthcare, USA). cDNA was constructed using the TaqMan miRNA reverse transcription kit (DBI Bioscience, Germany). The TaqMan miRNA kit (DBI Bioscience, Germany) was applied to measure miRNA expression. miR-421 expression was normalized to U6 using the 2^*−ΔΔCt*^ method.

The following primers were designed:

Induced nitric oxide synthase (iNOS): F: 5′-GCCACCAACAATGGCAACAT-3′, R: 5′-AGCAAAGAGGACTGTGGCTC-3′;

Interleukin (IL)-6: F: 5’-CACTTCACAAGTCGGAGGCT-3’, R: 5′-GCCACTCCTTCTGTGACTCC-3′;

Tumor necrosis factor alpha (TNF-α): F: 5’-CACTCTTCAGGGAACCAGGC-3’, R: 5′- GAGTTAGCTAGGCCACCCCA-3′;

Arginase 1 (Arg1): F: 5’-GTCCTTAAGCCGTTCCCTCG-3’, R: 5′-CGGCTGTGCATCATACAACG-3′;

Resistin-like molecule alpha 1 (Fizz1): F: 5’-AGGCCTGCATCAGTCTATGG-3’, R: 5′-TGGGTGATAAAACATGAAACCAAGT-3′;

miR-421: F: 5’-CTCACTCACATCAACAGACATTAATT-3’, R: 5′-GTGCAGGGTCCGAGGT-3′;

GAPDH: F: 5′- GCATCTTCTTGTGCAGTGCC-3′, R: 5′-ACTGTGCCGTTGAATTTGCC-3′; and.

U6: F: 5′- CTCGCTTCGGCAGCACATATACT-3′, R: 5′- ACGCTTCACGAATTTGCGTGTC-3′.

### Western blot

Total proteins from the exosomes or cells were extracted and detected using the bicinchoninic acid kit. Briefly, 40 µg of total protein was separated using sodium dodecyl-sulfate polyacrylamide gel electrophoresis (120 V, 90 min) and transferred to polyvinylidene fluoride (PVDF) membranes (90 V, 90 min). For blocking, 5% non-fat milk was added to the PVDF membranes for 1 h. Anti-CD9 (1: 1,000, ab307085, Abcam, Cambridge, MA, USA), anti-CD63 (1: 1,000, ab217345, ab68418), anti-CD81 (1: 1,000, ab109201), anti-SIRT3 (1: 500, ab189860), anti-AMPK (1: 1,000, ab207442), anti-p-AMPK (1: 1,000, ab133448), anti-LC3II/I (1: 1,000, ab48394), anti-P62 (1: 1,000, ab240635), anti-NLRP3 (1: 1,000, ab263899), anti-apoptosis-associated speck-like protein containing a CARD domain (ASC) (1: 1,000, ab70627), anti-IL-1β (1: 1,000, ab283822), anti-iNOS (1:1,000, ab178945), anti-IL-6 (1: 1,000, ab233706), anti-TNF-α (1:1,000, ab183218), anti-Calnexin (1:1000, ab22595) and anti-GAPDH (1:1,000, ab8245) were added and incubated overnight at 4 °C. Moreover, the horseradish peroxidase-labeled goat-anti-rabbit secondary antibody (1:5,000 diluted) was incubated at 25 ℃ for 2 h. Protein blots were detected using Pierce™ ECL (Thermo Fisher, Waltham, USA) in ChemiDoc MP (Bio-Rad, California, USA).

### Statistical analysis

Data are expressed as mean ± SD. All statistical analyses were performed using the one-way analysis of variance and Tukey’s multiple comparison tests (GraphPad Prism version 7.0). T test is conducted to analyze the differences between the groups. *P*-values < 0.05 indicated statistical significance.

## Results

### OSA-derived exosomes promote hepatocyte steatosis in mice

**Fig. 1:**
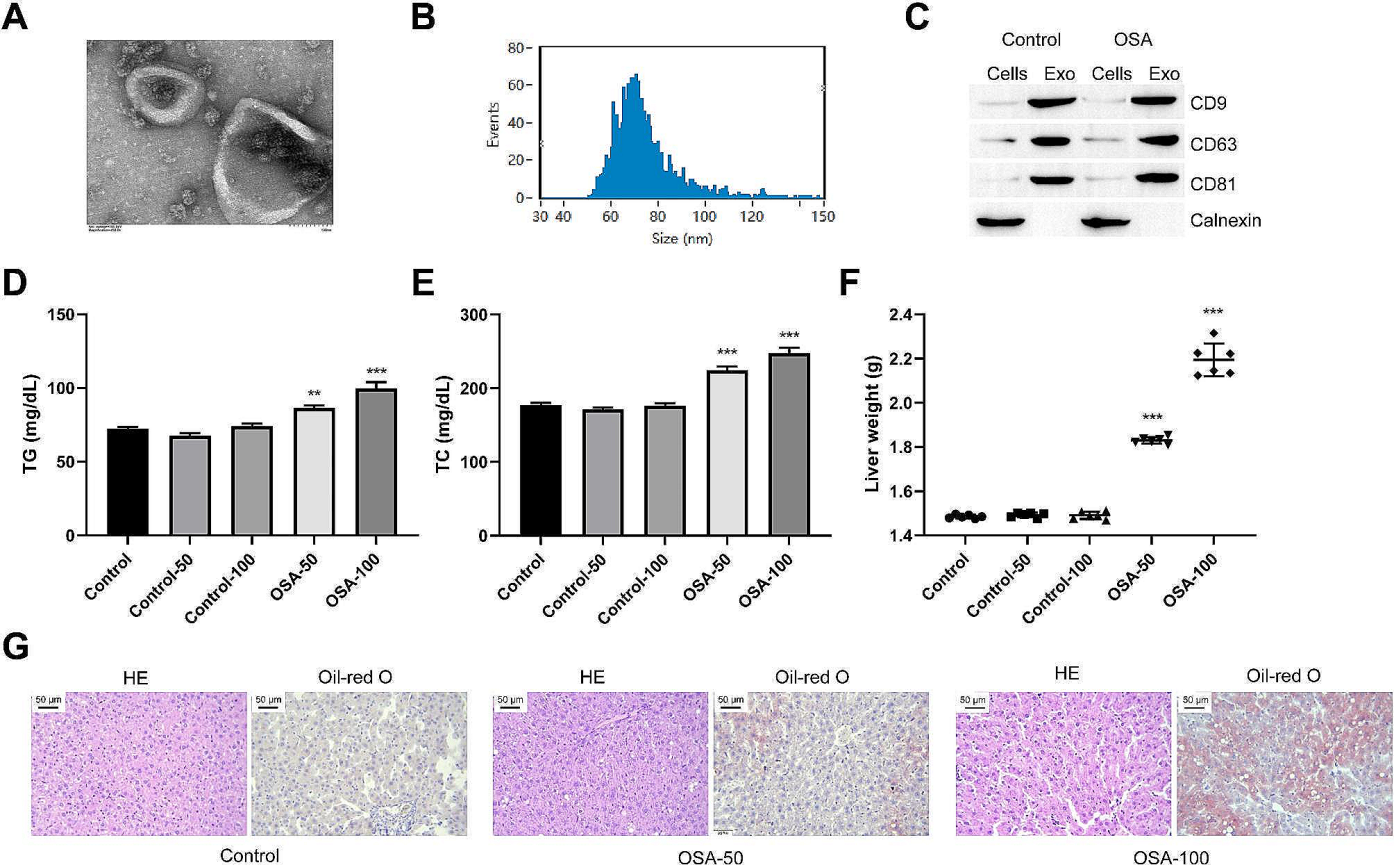
Exosomes from OSA mice promote hepatocyte steatosis. Exosomes derived from healthy mice and exosomes derived from OSA mice were injected into healthy mice (intraperitoneal injection, 50–100 µg/mouse). **(A)** The isolated exosomes were observed by TEM. **(B)** The size of the isolated exosomes was detected by a particle size analyzer. **(C)** Exosomal marker proteins CD9, CD63 and CD81 were detected via Western blot. **(D-E)** The effect of OSA-derived exosomes on the levels of cholesterol and triglycerides (TG) in peripheral blood of mice. **(F)** The effect of OSA-derived exosomes on the liver weight. **(G)** The effects of OSA-derived exosomes on liver tissue damage and fat accumulation were detected by HE staining and Oil Red O staining, respectively. ^*^P < 0.05, ^**^P < 0.01, ^***^P < 0.001 vs. Control group

To analyze the role of OSA-related exosomes in NAFLD, the OSA mice model was induced via intermittent hypoxia. Exosomes were collected from the peripheral blood of mice. The diameter of exosomes primarily ranged between 50 nm and 150 nm (Fig. 1A and B). The expression of exosomal marker factors CD9, CD63, and CD81 were noticeable, and the level of the negative marker protein Calnexin in exosomes was almost non-existent (Fig. 1C). A similar method was used to collect exosomes from healthy mice. Exosomes derived from healthy and OSA mice were injected intraperitoneally into healthy mice (50 µg/mouse or 100 µg/mouse). After 6 weeks, we observed no apparent changes in the TC and TG levels in the peripheral blood of mice that received exosomes from healthy sources (Fig. 1D and E). Moreover, the liver mass did not change significantly (Fig. 1F). TC and TG levels in the peripheral blood of mice receiving OSA-derived exosomes increased significantly. Additionally, the liver mass increased in a dose-dependent manner (Fig. 1D and F). Histological examination confirmed that after the intervention, the hepatocytes were stained unevenly. Moreover, inflammatory infiltration and fat accumulation appeared (Fig. 1G). Thus, OSA-derived exosomes could cause fat accumulation in the liver tissue, suggesting that exosomes in OSA may be an important factor in inducing NAFLD.

### OSA-derived exosomes promote hepatocyte fat accumulation by promoting macrophage M1 polarization

**Fig. 2:**
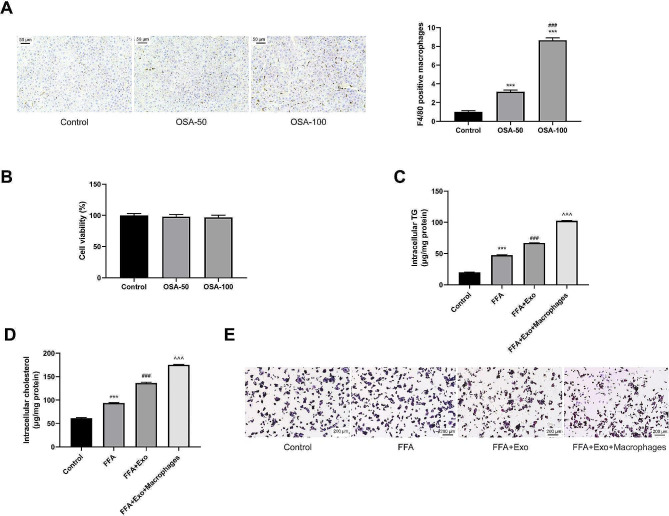
OSA-derived exosomes promote hepatocyte fat accumulation by promoting macrophage M1 polarization. Exosomes derived from OSA mice were injected into healthy mice (intraperitoneal injection, 50–100 µg/mouse). **(A)** Immunofluorescence staining was used to detect F4/80 to analyze the activated macrophages in liver tissues. Hepatocytes were divided into control group, FFA group, FFA + Exo group, FFA + Exo + Macrophages group. FFA group: FFA was used to induce NAFLD in vitro; FFA + Exo: 20 µg/mL OSA-derived exosomes was added; FFA + Exo + Macrophages: A co-culture model of hepatocytes and macrophages was constructed, and then FFA and exosomes were added. **(B)** CCK-8 was used to detect the effect of Exo on cell viability. **(C-D)** The levels of cholesterol and triglycerides (TG) in the cells were compared. **(E)** Oil red O staining was used to detect steatosis of hepatocytes. ^*^P < 0.05, ^**^P < 0.01, ^***^P < 0.001 vs. Control group; ^#^*P* < 0.05, ^##^*P* < 0.01, ^###^*P* < 0.001 vs. FFA group; ^^^*P* < 0.05, ^^^^*P* < 0.01, ^^^^^*P* < 0.001 vs. FFA + Exo group

Macrophage activation and M1 polarization are the key to inducing NAFLD. We analyzed the association between the effect of OSA-derived exosomes in promoting steatosis and macrophages. First, we detected macrophages in the liver tissues. We detected F4/80 using immunofluorescence staining to analyze the activated macrophages. OSA-derived exosomes promoted macrophage activation in the liver tissues of healthy mice (Fig. 2A). Different exosome doses were used to treat the cells; they exerted no significant effect on cell viability (Fig. 2B).

Second, the hepatocytes were divided into the control, FFA, FFA + Exo, and FFA + Exo + Macrophages groups. In the FFA group, we used FFA to induce NAFLD in vitro. In the FFA + Exo group, we used 20 µg/mL of OSA-derived exosomes and FFA simultaneously. In the FFA + Exo + Macrophages group, we constructed a co-culture model of hepatocytes and macrophages. Subsequently, FFA and exosomes were added. FFA moderately induced hepatocyte steatosis and increased the TG and TC levels in the hepatocytes. The addition of exosomes promoted fat accumulation. More importantly, steatosis was more obvious in the presence of macrophages; fat accumulation increased further (Fig. 2C and E). Thus, the macrophages were involved in NAFLD induced by OSA-derived exosomes.

### OSA-derived exosomes promote macrophage activation and M1 polarization

**Fig. 3:**
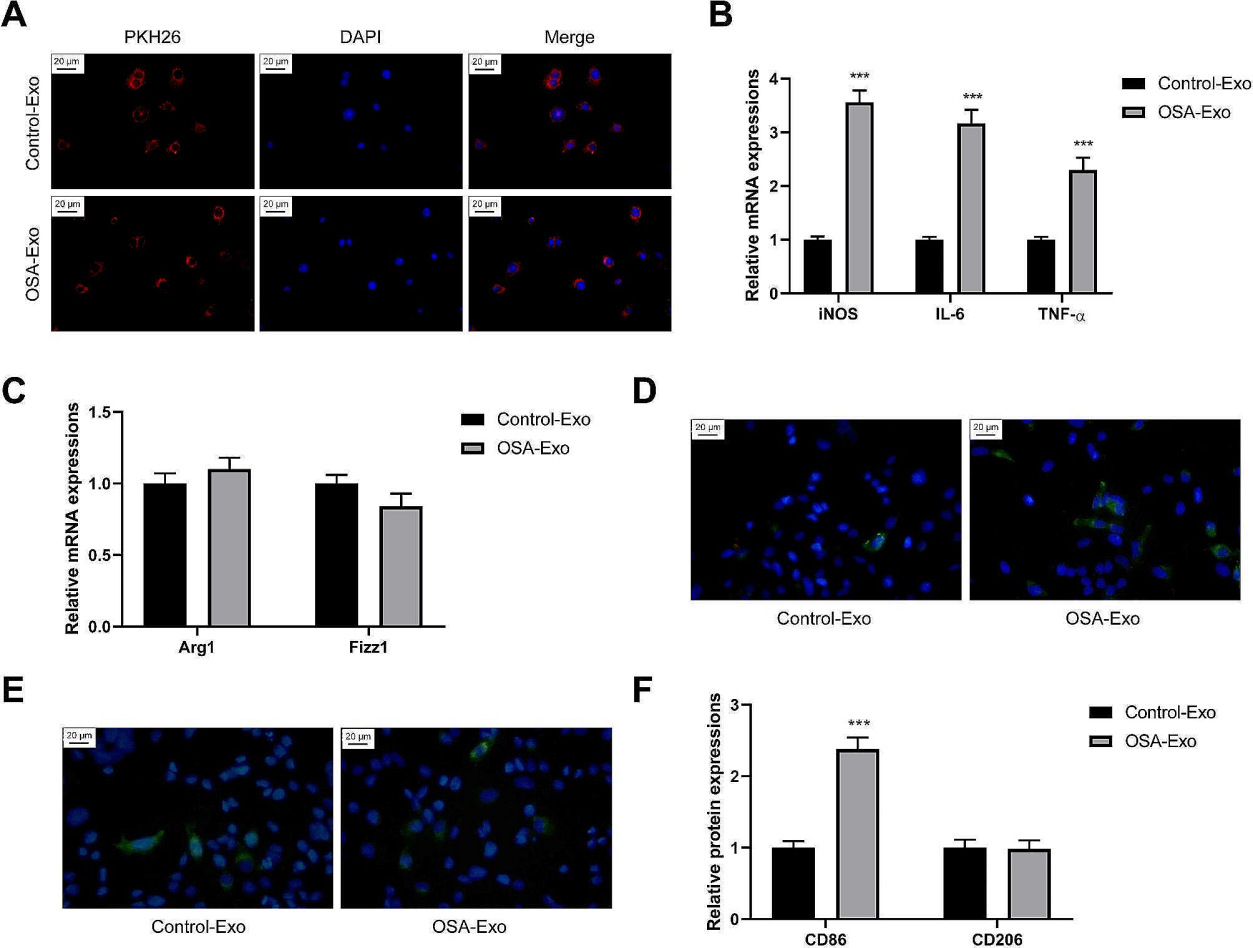
OSA-derived exosomes promote macrophage activation and M1 polarization. **(A)** The exosomes were labeled with PKH26 (red) and the nucleus were labeled with DAPI, the internalization of exosomes by macrophages was observed. Exosomes from healthy mice and OSA mice were used to stimulate macrophages (20 µg/mL). **(B)** The polarization level of M1 macrophages was analyzed by detecting the expression levels of iNOS, IL-6, and TNF-α mRNA via RT-qPCR. **(C)** The polarization level of M2 macrophages was analyzed by detecting the expression levels of Arg1 and Fizz1 mRNA via RT-qPCR. **(D, E, F)** The percentages of M1 and M2 macrophages after induction were detected by IHF. (Green in Fig. 3D: CD86, Blue in Fig. 3D: DAPI; Green in Fig. 3E: CD206, Blue in Fig. 3E: DAPI). ^*^P < 0.05, ^**^P < 0.01, ^***^P < 0.001 vs. control-Exo group

Exosomes from healthy and OSA mice were used to stimulate the macrophages (20 µg/mL). They were labeled using PKH26 (red), and the nucleus was stained using DAPI (blue). The macrophages could internalize the exosomes (Fig. 3A). After internalization of the OSA-derived exosomes, the transcription levels of M1 type marker genes of macrophages, namely iNOS, IL-6, and TNF-α, increased significantly. By contrast, the transcription levels of M2 type marker genes, namely Arg1 and Fizz1, did not change significantly (Fig. 3B and C). M1-type macrophages induced by OSA-derived exosomes displayed a higher proportion than those induced by healthy exosomes (Fig. 3D and F). The proportion of M2-type macrophages did not change significantly (Fig. 3E and F). Thus, OSA-derived exosomes could be internalized by macrophages and promote M1 polarization.

### OSA-derived exosomes inhibit the SIRT3/AMPK pathway in macrophages and autophagy and promote NLRP3 protein expression

**Fig. 4:**
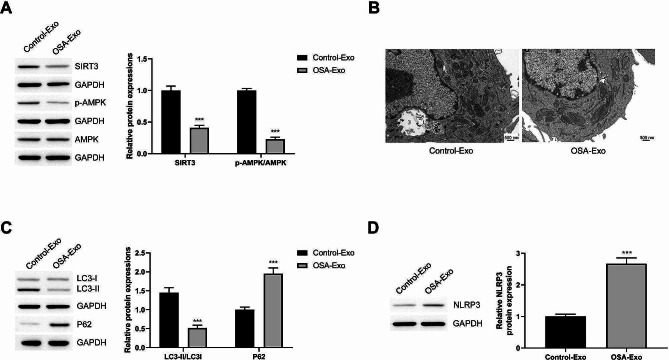
OSA-derived exosomes inhibit the SIRT3/AMPK pathway in macrophages, inhibit autophagy, and promote NLRP3 protein expression. Exosomes from healthy mice and OSA mice were used to stimulate macrophages (20 µg/mL). **(A)** The effects of OSA-derived exosomes on SIRT3 protein levels and AMPK protein phosphorylation levels. **(B)** TEM was used to observe the autophagosomes in macrophages. **(C)** The effects of OSA-derived exosomes on autophagy marker proteins LC3II/I and P62 were detected via Western blot. **(D)** The effects of OSA-derived exosomes on NLRP3 protein level were detected by Western blot. ^*^P < 0.05, ^**^P < 0.01, ^***^P < 0.001 vs. Control-Exo group

NLRP3 inflammasome is central to inducing M1 polarization. The SIRT3/AMPK pathway regulated the degradation of NLRP3 inflammasomes by regulating autophagy. To analyze the molecular mechanism by which OSA-derived exosomes promote macrophage polarization, we detected the SIRT3/AMPK pathway, autophagy levels, and NLRP3 protein levels. Compared with exosome induction from healthy mice, the use of OSA-derived exosomes induced SIRT3 protein down-regulation in macrophages and increased AMPK protein phosphorylation (Fig. 4A). No intracellular autophagosomes were observed in the macrophages induced by OSA-derived exosomes (Fig. 4B). The autophagosome marker protein LC3-II/I decreased, whereas the autophagy substrate protein P62 increased (Fig. 4C). Moreover, OSA-derived exosomes increased NLRP3 protein levels in the macrophages (Fig. 4D). Thus, OSA-derived exosomes may regulate autophagy through the SIRT3/AMPK pathway, thereby regulating NLRP3 expression (an M1 polarization factor) and inducing macrophage polarization.

### SIRT3/AMPK promotes NLRP3 inflammasome degradation by promoting autophagy and inhibits M1 polarization

**Fig. 5:**
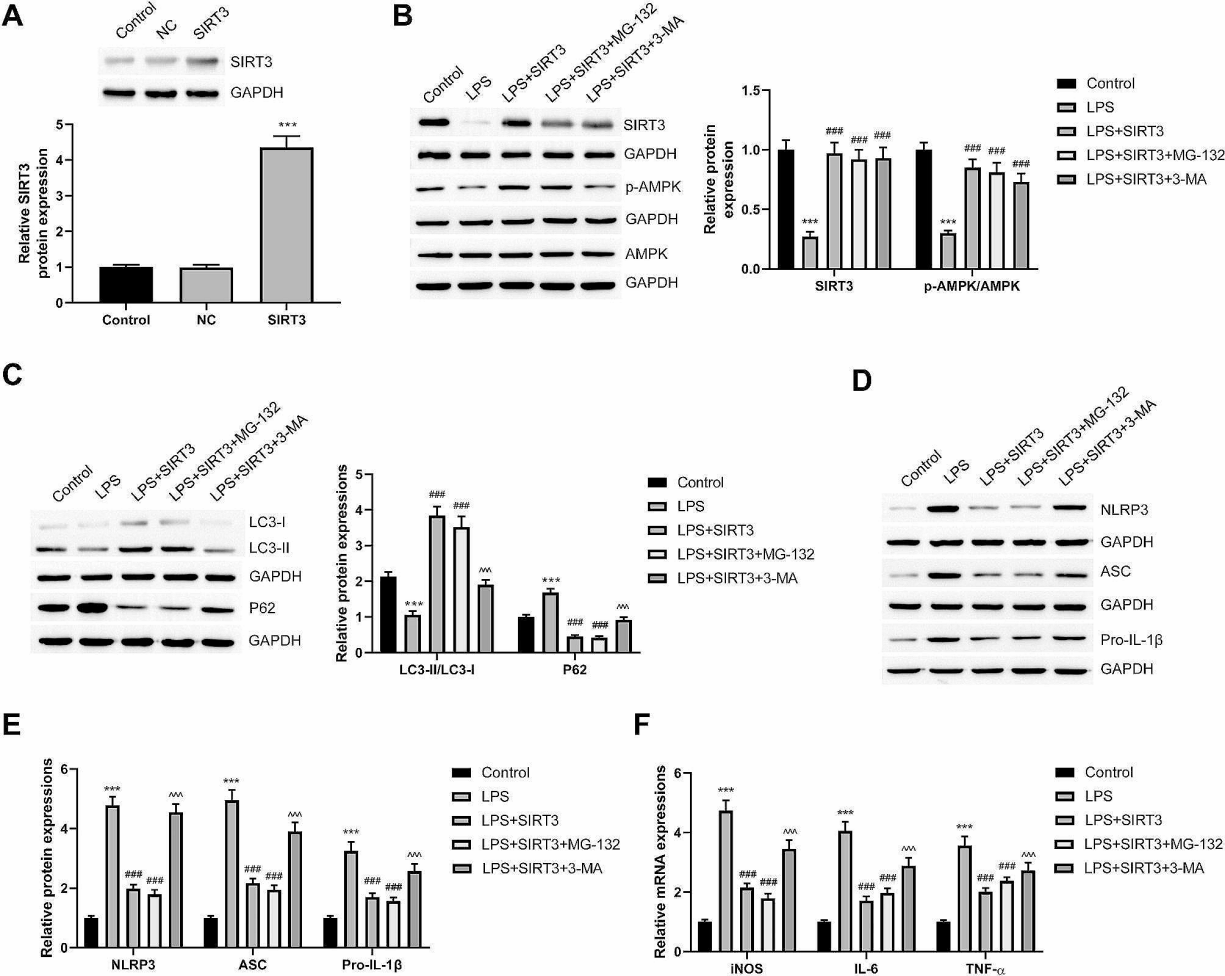
SIRT3/AMPK promotes the degradation of NLRP3 inflammasomes by promoting autophagy and inhibits M1 polarization of macrophages. **(A)** SIRT3 overexpressing macrophages are constructed. LPS was used to activate NLPR3 inflammasome and induced M1 polarization of macrophages. MG-132 and 3-MA were applied to inhibit proteasome and autophagy, respectively. **(B)** The SIRT3 protein expression and the phosphorylation levels of AMPK protein in the cells of each group were compared via Western blot. **(C)** The autophagy levels of each group were compared, autophagy marker proteins LC3II/I and P62 were detected via Western blot. **(D, E)** The activation of NLRP3 inflammasomes in each group was detected by Western blot. **(F)** The expression levels of iNOS, IL-6, and TNF-α mRNA in each group was compared via RT-qPCR. ^*^P < 0.05, ^**^P < 0.01, ^***^P < 0.001 vs. Control group; ^#^*P* < 0.05, ^##^*P* < 0.01, ^###^*P* < 0.001 vs. LPS group; ^^^*P* < 0.05, ^^^^*P* < 0.01, ^^^^^*P* < 0.001 vs. LPS + SIRT3 group

SIRT3 was overexpressed in the macrophages (Fig. 5A). We used LPS to activate NLPR3 inflammasomes and induce the M1 polarization of macrophages. The proteasome inhibitor MG-132 was used to inhibit ubiquitination degradation. Moreover, 3-MA was used to inhibit autophagy in the cells. SIRT3 overexpression promoted the phosphorylation and activation of AMPK (Fig. 5B). Additionally, it inhibited the levels of NLRP3 inflammasome constituent proteins, namely NLRP3 and ASC, and reduced IL-1β production (Fig. 5C and E). Moreover, SIRT3 overexpression significantly alleviated LPS-induced iNOS, IL-6, and TNF-α mRNA expressions (Fig. 5D). MG-132 and 3-MA exerted no significant effect on SIRT3 protein levels and AMPK protein phosphorylation (Fig. 5B). Proteasome inhibition by MG-132 did not significantly affect autophagy in the cells (Fig. 5C). Correspondingly, NLRP3 inflammasome activation and M1 polarization did not change significantly (Fig. 5D and F). However, the use of 3-MA to inhibit autophagy flux restored NLRP3 inflammasome activity. Furthermore, it increased the transcription level of M1-type marker genes (Fig. 5C and F). Autophagy inhibition blocked the effects of SIRT3 in promoting NLRP3 degradation and inhibiting M1 polarization. Thus, in liver tissue-derived macrophages, SIRT3 promoted autophagy by activating AMPK, thereby inducing the autophagy and degradation of NLRP3 inflammasomes. Eventually, it inhibited M1 polarization.

### Exosomes inhibit SIRT3 protein expression in macrophages by delivering miR-421

Exosomes facilitate miRNA delivery and can protect miRNA from degradation. The upstream miRNAs of SIRT3 were predicted through three tool websites. A Venn diagram was constructed, and the following six miRNAs were identified: miR-525-3p, miR-3139, miR-3150b-3p, miR-4784, miR-524-3p, and miR-421 (Figure S1A). Changes were detected in these six miRNAs in the exosomes of healthy, OSA, and OSA + NAFLD-derived mice and patients with OSA. miR-421 levels were significantly increased in the OSA-derived exosomes of mice. Moreover, miR-421 levels were further increased in OSA + NAFLD mice- and patient-derived exosomes (Figure S1B). miR-421 levels were noticeably higher in the circulating exosomes of patients with OSA than in healthy people. Additionally, miR-421 levels were significantly higher in the exosomes of patients with OSA and NAFLD than in patients with OSA (Figure S1C). The dual luciferase report confirmed the targeted binding of miR-421 to the 3’-UTR of SIRT3 mRNA in the macrophages (Figure S1D-E). miR-421 displayed similar co-localization with macrophages in the liver tissues of OSA and OSA + NAFLD mice (Figure S1F). Moreover, miR-421 mimic transfection increased miR-421 levels in macrophages (Figure S1G). miR-421 overexpression significantly inhibited SIRT3 protein expression (Figure S1H). Thus, miR-421 in exosomes may inhibit SIRT3 expression in macrophages.

### Intermittent hypoxia-induced hepatocytes deliver miR-421 to the macrophages via exosomes to inhibit SIRT3

First, hepatocytes in the OSA environment were simulated using intermittent hypoxia. Exosomes secreted by the hepatocytes were collected and identified using exosomal marker proteins (CD9, CD63, and CD81) and hepatocyte marker protein (CYP2E1) (Figure S2A). After inducing intermittent hypoxia, miR-421 levels increased in the hepatocytes and secreted exosomes (Figure S2B). Imaging experiments confirmed that the secreted exosomes were internalized by macrophages (Figure S2C). Transfection of miR-421 inhibitor to inhibit miR-421 in macrophages (Figure S2D). Thus, miR-421 inhibition alleviated LPS-induced M1 polarization (Figure S2E,F).

**Fig. 6:**
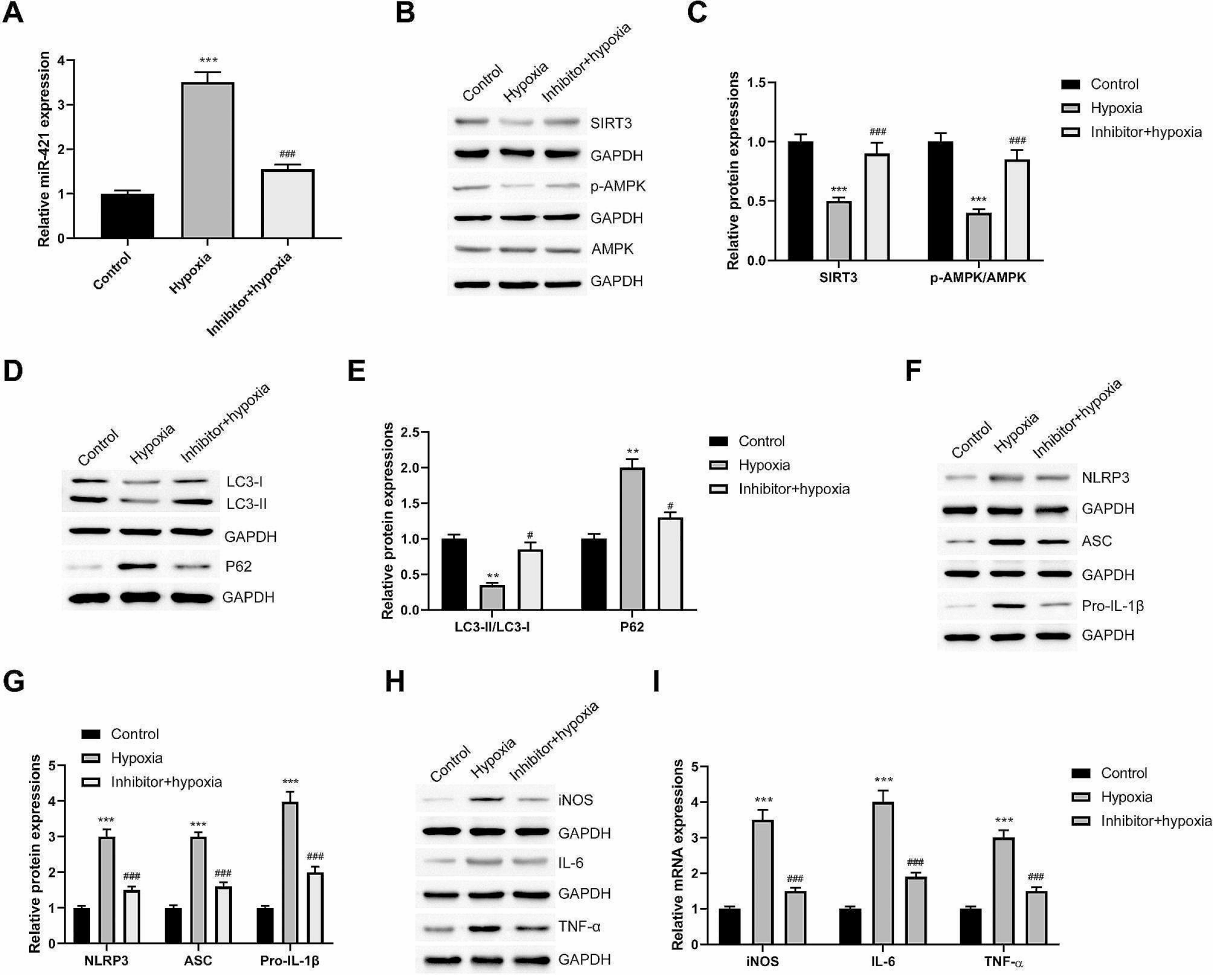
Intermittent hypoxia-induced hepatocytes deliver miR-421 to macrophages via exosomes to inhibit SIRT3, thereby participating in macrophage M1 polarization. Macrophages were divided into 3 groups: control, inhibitor, hypoxia, and inhibitor + hypoxia. Intermittent hypoxia induced hepatocyte-derived exosomes were used to induce normal macrophages and macrophages transfected with miR-421 inhibitor. **(A)** RT-qPCR was used to compare the miR-421 levels in macrophages. **(B, C)** Effects of exosomes secreted by hepatocytes induced by intermittent hypoxia and miR-421 inhibitor on SIRT3/AMPK pathway in macrophages via Western blot. **(D, E)** Western blot was applied to test the autophagy levels in macrophages in each group. **(F, G)** Comparison of NLRP3 inflammasome in macrophages in each group via Western blot. **(H, I)** Western blot and RT-qPCR were used to detect the M1 polarization marker proteins and mRNA (iNOS, IL-6 and TNF-α) in macrophages in each group. ^*^P < 0.05, ^**^P < 0.01, ^***^P < 0.001 vs. Control group; ^#^*P* < 0.05, ^##^*P* < 0.01, ^###^*P* < 0.001 vs. Hypoxia group

To confirm the correlation between miR-421 and hypoxia-induced hepatocyte exosomes promoting M1 polarization, the macrophages were divided into three groups as follows: control, inhibitor, hypoxia, and inhibitor + hypoxia groups. Intermittent hypoxia-induced hepatocyte-derived exosomes were used to induce healthy and miR-421 inhibitor-transfected macrophages. First, macrophages could internalize the exosomes secreted by hepatocytes induced by intermittent hypoxia, which could lead to miR-421 upregulation (Fig. 6A). These exosomes inhibited SIRT3 protein expression and AMPK protein phosphorylation in macrophages. Additionally, they inhibited autophagy and promoted NLRP3 inflammasome activation. Suppressing miR-421 levels in macrophages significantly alleviated the inhibitory effects of exosomes on SIRT3/AMPK and autophagy as well as the promotion of NLRP3 inflammasomes (Fig. 6B and G). Additionally, miR-421 inhibition significantly blocked the function of exosomes to promote M1 polarization (Fig. 6H and I). Thus, the promotion of M1 polarization by hypoxia-induced hepatocyte exosomes was inseparable from miR-421.

### Exosomes-mediated miR-421 participates in macrophage polarization to promote NAFLD in vivo

**Fig. 7:**
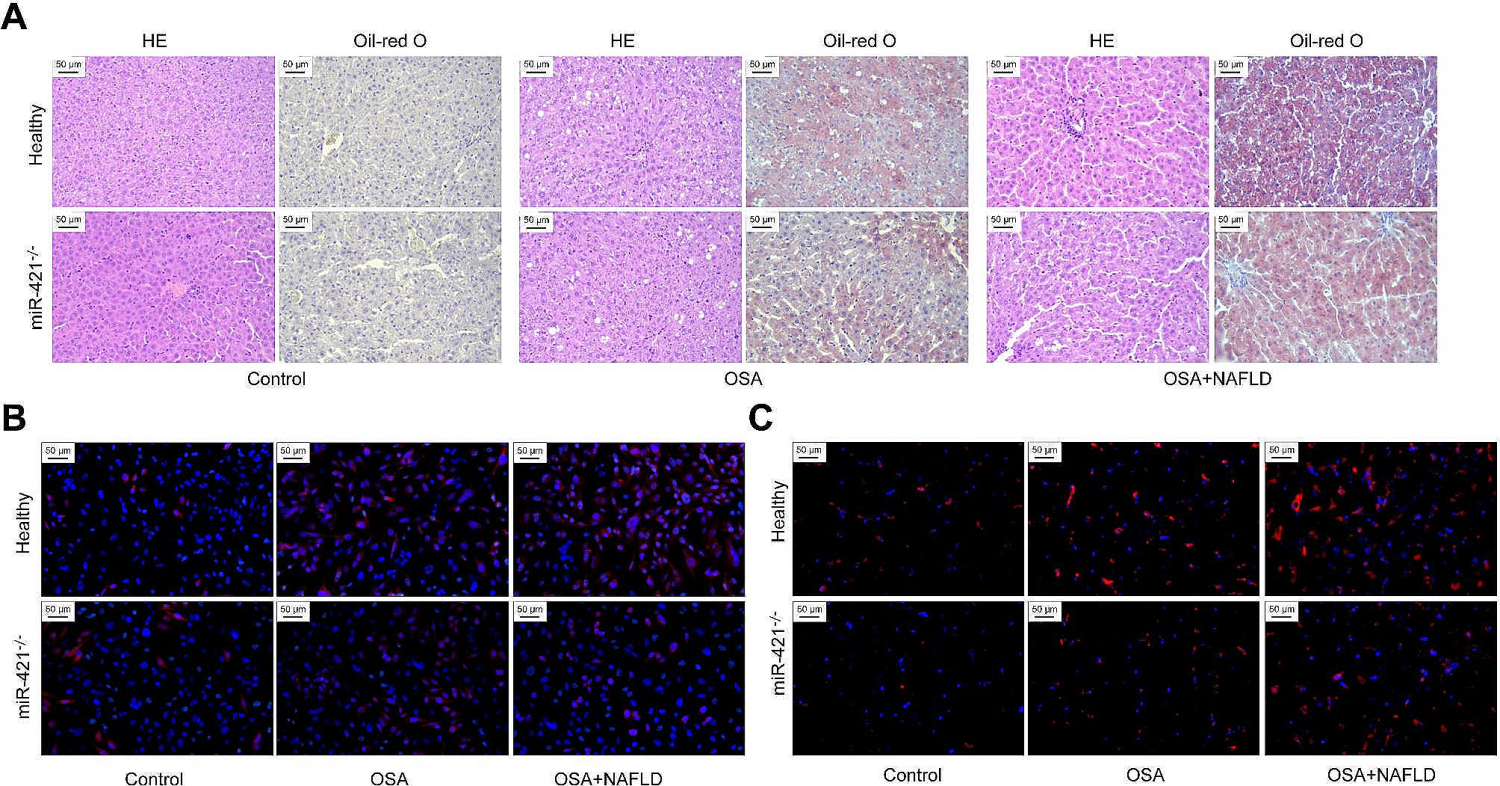
Exosomes-mediated miR-421 participates in macrophage polarization to promote NAFLD in vivo. Normal mice and miR-421^−/−^ mice were used to construct OSA and OSA + NAFLD models, respectively. **(A)** The effects of miR-421 lack on liver tissue damage and steatosis in OSA and OSA + NAFLD model mice were detected by HE and Oil red O staining. **(B)** MiR-421 fluorescence in situ hybridization (green) and F4/80 immunofluorescence staining (red) were used to analyze the co-localization of the two in liver tissues. **(C)** The levels of CD68-positive M1 macrophages in liver tissues were observed by IF staining

To confirm the role of miR-421 in OSA-related NAFLD, normal and miR-421^−/−^ mice were used to construct the OSA and OSA + NAFLD models, respectively. In the absence of miR-421, liver tissue damage and fat accumulation were significantly reduced in the OSA and OSA + NAFLD model mice (Fig. 7A). In liver tissues, miR-421 absence was associated with a decrease in activated macrophages (Fig. 7B and C). These findings confirmed the importance of miR-421 in OSA-induced liver steatosis in vivo.

### Exosome-mediated miR-421 regulates autophagy and promotes macrophage M1 polarization through SIRT3

**Fig. 8:**
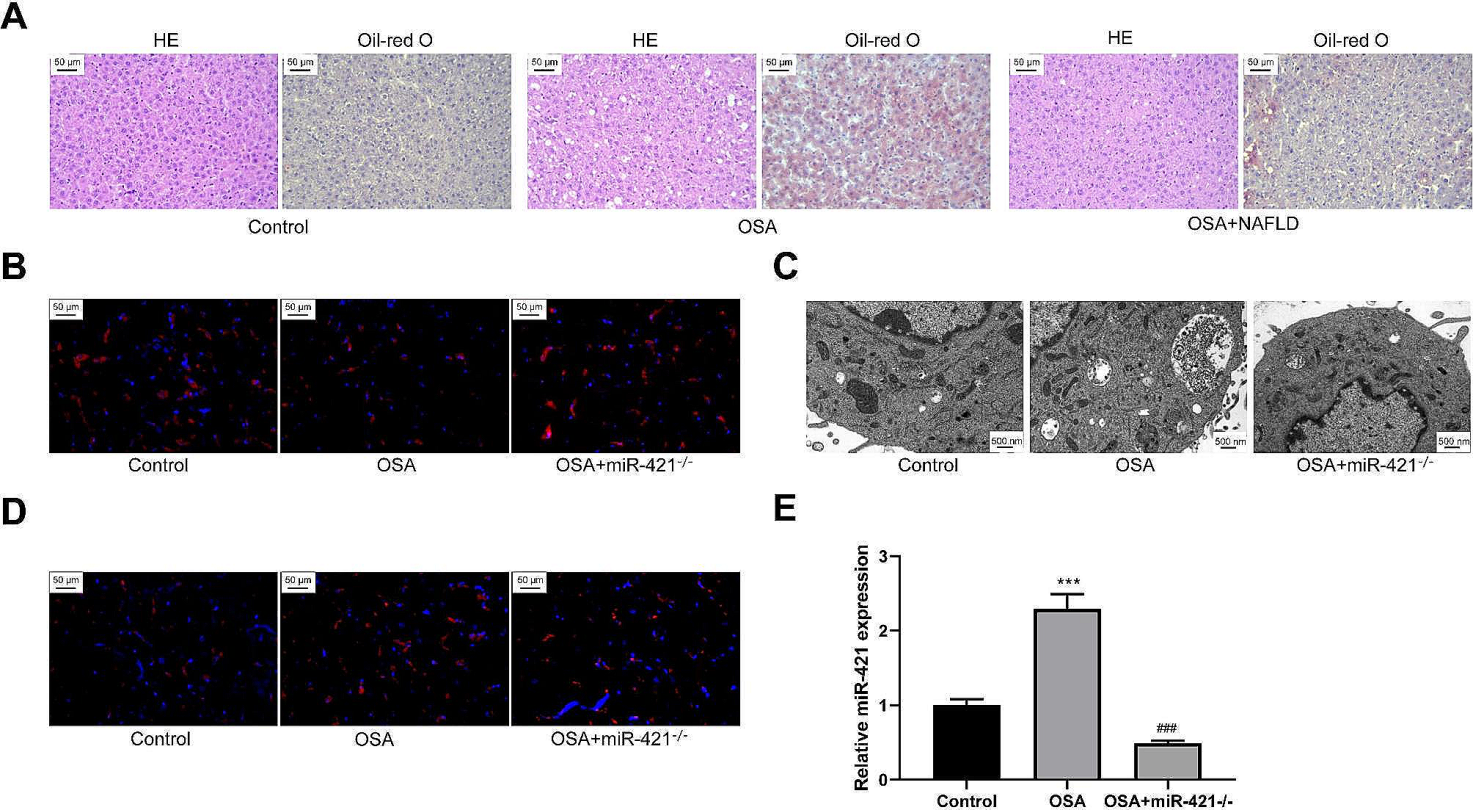
MiR-421 in OSA-derived exosomes promotes macrophage M1 polarization through regulating SIRT3 and autophagy in vivo. The exosomes from healthy mice, OSA mice, and miR-421^−/−^ OSA mice were used to treat healthy mice. **(A)** The effects of OSA-derived exosomes lacking in miR-421 on liver tissue damage and steatosis were detected by HE and Oil red O staining. **(B)** IF staining was used to detect the level and distribution of SIRT3 protein. **(C)** TEM was used to observe the autophagosomes in macrophages. **(D)** The levels of CD68-positive M1 macrophages in liver tissues were observed by IF staining.  ***P < 0.001 vs. Control group; ###*P* < 0.001 vs. OSA group

To verify the effects of miR-421 on macrophage polarization through SIRT3 in vivo, the mice were divided into three groups as follows: control, OSA, and OSA + miR-421^−/−^ groups. Exosomes from healthy, OSA, and miR-421^−/−^ OSA mice were used to treat healthy mice. The abilities of miR-421^−/−^ OSA mice-derived exosomes to induce liver tissue damage and fat accumulation were significantly blocked (Fig. 8A). Compared with the control group, SIRT3 protein levels significantly increased in the liver tissues of the OSA group. The absence of miR-421 restored SIRT3 protein levels in macrophages of liver tissues (Fig. 8B). Moreover miR-421 suppression restored the autophagy suppression of macrophages induced by OSA-derived exosomes (Fig. 8C). Additionally, OSA-derived exosomes increased in miR-421 and augmented M1 macrophages in liver tissues. The promotion effect of miR-421^−/−^ OSA mice-derived exosomes was significantly weakened on M1-type macrophages (Fig. 8D and E). Thus, exosomes could inhibit SIRT3 and autophagy by delivering miR-421 to macrophages and promote M1 polarization in vivo, thereby participating in OSA-induced liver steatosis.

## Discussion

OSA induces hepatocytes to secrete exosomes containing excessive miR-421, which can participate in NAFLD by promoting the M1 proliferation of macrophages. These exosomes are internalized by macrophages. Subsequently, miR-421 targets and inhibits the SIRT3/AMPK pathway in macrophages, thereby inhibiting autophagy and promoting NLRP3 inflammasome activation. This phenomenon promotes macrophage activation and leads to liver steatosis. Therefore, miR-421 detection in exosomes may predict complications in patients with OSA. Therefore, targeting miR-421 may be an effective strategy to alleviate OSA with NAFLD.

OSA can induce numerous systemic complications, such as NAFLD. Exosomes may serve as the link between OSA and NAFLD. Exosomes facilitate transmitting information between cells. Almost all cells can secrete exosomes, which regulate the target cells through changes in the miRNA, mRNA, and protein levels contained therein. OSA induces several complications through exosomes, including erectile dysfunction [29], endothelial dysfunction [30, 31], insulin resistance [13, 32], abnormal blood pressure [33], and increased risk of tumors [34]. Exosomes are involved in NAFLD; nonetheless, the role of OSA-related exosomes in liver steatosis is unclear. The exosomes released by palmitic acid-stimulated hepatocytes can promote the activation of hepatic stellate cells, thereby promoting NAFLD and inducing liver fibrosis [35]. Myeloid cells deliver miR-223 to liver tissues through exosomes to alleviate NALFD fibrosis [36]. A high-fat diet affects the AMPK pathway in adipocytes and promotes exosome secretion, thereby inducing liver steatosis [37]. Therefore, we primarily explored whether OSA-related exosomes can cause NAFLD. First, the OSA mouse model was constructed by inducing intermittent hypoxia. Second, the exosomes were collected and identified. Exosomes derived from healthy and OSA mice were injected into healthy mice. OSA-derived exosomes significantly induced liver tissue damage and hepatocyte steatosis. Thus, OSA-derived exosomes are one of the mechanisms involved in NAFLD. Additionally, macrophages are significantly affected by exosomes. For example, stem cell-derived exosomes can alleviate inflammatory response and obesity by promoting M2 polarization of macrophages [38]. The OSA-related metabolic dysfunction is inseparable from macrophage regulation by exosomes [39]. F4/80-positive activated macrophage levels increased in fatty liver tissues induced by OSA-derived exosomes. In vitro experiments suggested that OSA-derived exosomes moderately promoted fat accumulation in hepatocytes. Moreover, macrophages promoted hepatocyte steatosis. Thus, macrophages are involved in NAFLD caused by OSA exosomes. This necessitated exploring the mechanism by which OSA-derived exosomes affect macrophages.

First, PKH26-labeled exosomes were internalized by the macrophages. After internalizing OSA-derived macrophages, the transcription levels of iNOS, IL-6, and TNF-α increased significantly. Furthermore, the proportion of M1-type macrophages is increased. We observed no significant change in the proportion of M2 macrophages. Compared with exosomes derived from healthy mice, those derived from OSA mice significantly inhibited SIRT3 protein expression and AMPK phosphorylation, inhibited autophagy, and activated NLRP3 inflammasomes. The SIRT3/AMPK pathway promotes autophagy [40–42]. In macrophages, SIRT3/AMPK can inhibit activation [16] and promote autophagy flux [43]. To confirm the role of SIRT3 and autophagy in OSA-related NAFLD, we used LPS to activate NLRP3 inflammasomes and induce macrophage M1 polarization. SIRT3 overexpression significantly activated AMPK and increased autophagy flux. Moreover, it inhibited the NLRP3 inflammasome activation. The proteasome inhibitor MG-132 exerted no noticeable effects. The autophagy inhibitor 3-MA significantly blocked NLRP3 degradation and the effect of SIRT3 on macrophage activation. Therefore, the components of OSA-derived exosomes may increase the level of NLRP3 inflammasomes by inhibiting the SIRT3/AMPK pathway in macrophages, thereby promoting M1 polarization and liver steatosis.

We identified the exosome components that affect the SIRT3/AMPK pathway in macrophages, based on the principle of base complementary pairing. miRNAs with binding sites for SIRT3 were searched from three websites. We detected changes in six miRNAs in the exosomes of patients with OSA, healthy, OSA, and OSA + NAFLD-derived mice. miR-421 levels were significantly increased in OSA-derived exosomes. miR-421 levels were further increased in OSA + NAFLD mice and patient-derived exosomes. SIRT3 levels are suppressed in liver tissues of the NAFLD model induced by a high-fat diet. miR-421 overexpression can target SIRT3 to aggravate mitochondrial dysfunction [44]. During liver fibrosis caused by hepatitis B virus, the level of miR-421 increases [45]. Additionally, miR-421 exerts a strong inhibitory effect on autophagy, including neurons [46, 47] and cardiomyocytes [48]. miR-421 can target SIRT3 mRNA and inhibit SIRT3 protein expression. Additionally, miR-421 and macrophages display similar co-localization in the liver tissues of OSA and OSA + NAFLD mice. Therefore, the miR-421 in OSA-derived exosomes can be internalized into macrophages, thereby regulating SIRT3 protein expression.

An in vitro study has demonstrated that the OSA environment can cause abnormalities in hepatocytes and secreted exosomes [49]. Hypoxia induces the release of exosomes [50]. The concentration of exosomes derived from hepatocytes is the highest in liver tissues. Under pressure, hepatocytes can release exosomes and cause changes in macrophages [20, 51]. Therefore, we analyzed whether OSA-induced hepatocytes can secrete miR-421-carrying exosomes and its effects on macrophages. miR-421 levels significantly increased in the hepatocytes and exosomes under intermittent hypoxia. Ge [52] reported that hypoxia induction can trigger miR-421 overexpression and promote gastric cancer metastasis and drug resistance. Additionally, in cardiomyocytes, the decrease in SIRT3 protein induced by hypoxia/reoxygenation is closely associated with the increase in miR-421 [53], consistent with our findings. An in vitro study confirmed that macrophages internalize exosomes derived from hepatocytes. Moreover, miR-421 inhibition in macrophages can block LPS-mediated M1-type activation. Additionally, exosomes secreted by hepatocytes under intermittent hypoxia inhibit the SIRT3/AMPK pathway and autophagy, promoting M1 polarization. Transfection with the miR-421 inhibitor significantly blocked the inhibitory effects of exosomes induced by intermittent hypoxia on SIRT3/AMPK and autophagy. Furthermore, its effect in promoting macrophage activation was significantly reduced. Thus, intermittent hypoxia hepatocytes can deliver numerous miR-421 to the macrophages through exosomes, thereby targeting and inhibiting the SIRT3/AMPK pathway, regulating autophagy, and promoting M1 polarization.

Additionally, we confirmed the role of miR-421 in NALFD caused by OSA in vivo. After modeling, miR-421^−/−^ mice demonstrated a milder degree of liver steatosis than healthy mice. Moreover, the number of M1-type macrophages in liver tissues was significantly reduced. miR-421^−/−^ mice-derived exosomes in the OSA model significantly relieved the induction of hepatic steatosis in healthy mice. Additionally, the absence of miR-421 blocked the inhibitory effects of mice-derived exosomes on SIRT3 and autophagy and alleviated the promotion of M1 polarization. Thus, OSA-mediated induction of NAFLD is inseparable from miR-421 at the in vivo level. In OSA, exosomes can regulate SIRT3/AMPK and autophagy in macrophages by delivering miR-421, thereby regulating macrophage polarization.

The effect of exosomes on hepatocyte steatosis is more noticeable in the presence of macrophages. Thus, we primarily analyzed the effect of exosomes on macrophages. However, in the OSA environment, numerous cells, including hepatocytes, were induced by intermittent hypoxia. In the OSA environment, exosomes can be transported to the entire body through the blood and may be received by all cells. Exosomes derived from brain tissues are internalized by macrophages through the blood-brain barrier. Therefore, the exosomes that affect macrophages do not originate only from hepatocytes. This study was only based on hepatocytes. The source of exosomes that affect macrophages in liver tissues during OSA warrants further investigation. The source, destination, and regulation of exosomes are complex and mutual in the internal environment.

## Conclusion

In conclusion, OSA-derived exosomes may regulate the SIRT3/AMPK pathway and autophagy by delivering miR-421 to macrophages, thereby promoting M1 polarization and inducing NAFLD. Thus, miR-421 may facilitate the diagnosis and treatment of OSA concurrent with NALFD.

### Electronic supplementary material

Below is the link to the electronic supplementary material.

Supplementary Material 1

Supplementary Material 2

## Data Availability

The authors declare that all the data supporting the findings in this study are available in this manuscript and its supplementary materials, or are available from the corresponding author through reasonable request.

## References

[1] Patel S.R (2019). Obstructive sleep apnea. Ann Intern Med.

[2] Jordan AS, McSharry DG, Malhotra A (2014). Adult obstructive sleep apnoea. Lancet.

[3] Siriwat R (2020). Obstructive sleep apnea and insulin resistance in children with obesity. J Clin Sleep Med.

[4] Gaines J (2018). Obstructive sleep apnea and the metabolic syndrome: the road to clinically-meaningful phenotyping, improved prognosis, and personalized treatment. Sleep Med Rev.

[5] Mesarwi OA, Loomba R, Malhotra A (2019). Obstructive sleep apnea, Hypoxia, and nonalcoholic fatty liver disease. Am J Respir Crit Care Med.

[6] Umbro I (2020). Association between non-alcoholic fatty liver disease and obstructive sleep apnea. World J Gastroenterol.

[7] Bhatt SP (2019). Non-alcoholic fatty liver disease is an independent risk factor for inflammation in obstructive sleep apnea syndrome in obese Asian indians. Sleep Breath.

[8] Zhang L (2020). Obstructive sleep apnea and liver injury in severely obese patients with nonalcoholic fatty liver disease. Sleep Breath.

[9] Peinado H (2016). Corrigendum: Melanoma exosomes educate bone marrow progenitor cells toward a pro-metastatic phenotype through MET. Nat Med.

[10] Kosaka N (2010). Secretory mechanisms and intercellular transfer of microRNAs in living cells. J Biol Chem.

[11] Rani S (2011). Isolation of exosomes for subsequent mRNA, MicroRNA, and protein profiling. Methods Mol Biol.

[12] Stoorvogel W (2012). Functional transfer of microRNA by exosomes. Blood.

[13] Khalyfa A (2018). Sleep-disordered breathing, circulating exosomes, and insulin sensitivity in adipocytes. Int J Obes (Lond).

[14] Khalyfa A (2016). Circulating plasma Extracellular Microvesicle MicroRNA Cargo and endothelial dysfunction in children with obstructive sleep apnea. Am J Respir Crit Care Med.

[15] Liu W (2019). Tim-4 inhibits NLRP3 Inflammasome via the LKB1/AMPKα pathway in macrophages. J Immunol.

[16] Duan WJ (2016). A SIRT3/AMPK/autophagy network orchestrates the protective effects of trans-resveratrol in stressed peritoneal macrophages and RAW 264.7 macrophages. Free Radic Biol Med.

[17] Zhang T (2020). Small molecule-driven SIRT3-autophagy-mediated NLRP3 inflammasome inhibition ameliorates inflammatory crosstalk between macrophages and adipocytes. Br J Pharmacol.

[18] Verma VK (2016). Alcohol stimulates macrophage activation through caspase-dependent hepatocyte derived release of CD40L containing extracellular vesicles. J Hepatol.

[19] Zhao Z (2020). Cholesterol impairs hepatocyte lysosomal function causing M1 polarization of macrophages via exosomal miR-122-5p. Exp Cell Res.

[20] Liu XL (2020). Lipotoxic hepatocyte-derived exosomal MicroRNA 192-5p activates macrophages through Rictor/Akt/Forkhead box transcription factor O1 signaling in nonalcoholic fatty liver disease. Hepatology.

[21] Teodorescu M (2023). Chronic intermittent hypoxia increases airway hyperresponsiveness during house dust mites exposures in rats. Respir Res.

[22] Bi H et al. Bone marrow stem cells therapy alleviates vascular injury in a chronic obstructive pulmonary disease–obstructive sleep apnea overlap syndrome rat model. Mol Med Rep, 2021. 23(1). 10.3892/mmr.2020.11707 PMC7716420 33236768

[23] Wu JG (2018). Effects of small interfering RNA targeting TLR4 on expressions of adipocytokines in obstructive sleep apnea hyponea syndrome with hypertension in a rat model. J Cell Physiol.

[24] Liu J (2017). Advanced Method for Isolation of Mouse Hepatocytes, Liver Sinusoidal endothelial cells, and Kupffer Cells. Methods Mol Biol.

[25] Valatas V (2003). Isolation of rat kupffer cells: a combined methodology for highly purified primary cultures. Cell Biol Int.

[26] Khalyfa A et al. Plasma exosomes in OSA patients promote endothelial senescence: effect of long-term adherent continuous positive airway pressure. Sleep, 2020. 43(2). 10.1093/sleep/zsz217 PMC7901815 31552414

[27] Rose KA (2016). Co-culture of hepatocytes and Kupffer Cells as an in vitro model of inflammation and Drug-Induced Hepatotoxicity. J Pharm Sci.

[28] Livak KJ, Schmittgen TD (2001). Analysis of relative gene expression data using real-time quantitative PCR and the 2(-Delta Delta C(T)) method. Methods.

[29] Liang L (2021). Exosomes derived from miR-301a-3p-overexpressing adipose-derived mesenchymal stem cells reverse hypoxia-induced erectile dysfunction in rat models. Stem Cell Res Ther.

[30] Khalyfa A (2016). Effect on intermittent hypoxia on plasma Exosomal Micro RNA signature and endothelial function in healthy adults. Sleep.

[31] Khalyfa A, Gozal D, Kheirandish-Gozal L. Plasma extracellular vesicles in children with OSA disrupt blood-brain Barrier Integrity and Endothelial Cell Wound Healing in Vitro. Int J Mol Sci, 2019. 20(24). 10.3390/ijms20246233 PMC6941040 31835632

[32] Khalyfa A (2021). Circulating exosomes and gut microbiome induced insulin resistance in mice exposed to intermittent hypoxia: effects of physical activity. EBioMedicine.

[33] Khalyfa A et al. Circulating plasma exosomes in obstructive sleep apnoea and reverse dipping blood pressure. Eur Respir J, 2020. 55(1). 10.1183/13993003.01072-2019 31672757

[34] Khalyfa A (2016). Circulating exosomes potentiate tumor malignant properties in a mouse model of chronic sleep fragmentation. Oncotarget.

[35] Lee YS (2017). Exosomes derived from palmitic acid-treated hepatocytes induce fibrotic activation of hepatic stellate cells. Sci Rep.

[36] Hou X (2021). Myeloid-cell-specific IL-6 signaling promotes MicroRNA-223-Enriched Exosome production to Attenuate NAFLD-Associated Fibrosis. Hepatology.

[37] Yan C (2021). A high-Fat Diet attenuates AMPK α1 in adipocytes to Induce Exosome Shedding and nonalcoholic fatty liver development in vivo. Diabetes.

[38] Zhao H (2018). Exosomes from adipose-derived stem cells attenuate adipose inflammation and obesity through polarizing M2 macrophages and beiging in White Adipose tissue. Diabetes.

[39] Khalyfa A, Kheirandish-Gozal L, Gozal D. Exosome and Macrophage Crosstalk in Sleep-Disordered Breathing-Induced metabolic dysfunction. Int J Mol Sci, 2018. 19(11). 10.3390/ijms19113383 PMC6274857 30380647

[40] Li Y (2021). Stilbene Glycoside upregulates SIRT3/AMPK to promotes neuronal mitochondrial autophagy and inhibit apoptosis in ischemic stroke. Adv Clin Exp Med.

[41] Han D (2020). SIRT3 deficiency is resistant to autophagy-dependent ferroptosis by inhibiting the AMPK/mTOR pathway and promoting GPX4 levels. J Cell Physiol.

[42] Zhao W (2018). SIRT3 protects against acute kidney Injury via AMPK/mTOR-Regulated autophagy. Front Physiol.

[43] Li Y, et al. LncRNA DYNLRB2-2 inhibits THP-1 macrophage foam cell formation by enhancing autophagy. Biol Chem; 2019. 10.1515/hsz-2018-0461 30903747

[44] Cheng Y (2016). MicroRNA-421 induces hepatic mitochondrial dysfunction in non-alcoholic fatty liver disease mice by inhibiting sirtuin 3. Biochem Biophys Res Commun.

[45] Orr C (2020). Longitudinal analysis of serum microRNAs as predictors of cirrhosis regression during treatment of hepatitis B virus infection. Liver Int.

[46] Wang J (2020). MicroRNA-421-3p-abundant small extracellular vesicles derived from M2 bone marrow-derived macrophages attenuate apoptosis and promote motor function recovery via inhibition of mTOR in spinal cord injury. J Nanobiotechnol.

[47] Hu F (2020). Knockdown of ZFAS1 inhibits hippocampal neurons apoptosis and autophagy by activating the PI3K/AKT pathway via Up-regulating miR-421 in Epilepsy. Neurochem Res.

[48] Zhang CL (2021). CircPAN3 ameliorates myocardial ischaemia/reperfusion injury by targeting miR-421/Pink1 axis-mediated autophagy suppression. Lab Invest.

[49] Hernández A (2020). Chemical hypoxia induces pro-inflammatory signals in fat-laden hepatocytes and contributes to cellular crosstalk with Kupffer cells through extracellular vesicles. Biochim Biophys Acta Mol Basis Dis.

[50] Yu X (2012). Mechanism of TNF-α autocrine effects in hypoxic cardiomyocytes: initiated by hypoxia inducible factor 1α, presented by exosomes. J Mol Cell Cardiol.

[51] Babuta M (2019). Dysregulated autophagy and lysosome function are linked to Exosome production by Micro-RNA 155 in alcoholic liver disease. Hepatology.

[52] Ge X (2016). MicroRNA-421 regulated by HIF-1α promotes metastasis, inhibits apoptosis, and induces cisplatin resistance by targeting E-cadherin and caspase-3 in gastric cancer. Oncotarget.

[53] Liu Y (2020). MiR-421 inhibition protects H9c2 cells against hypoxia/reoxygenation-induced oxidative stress and apoptosis by targeting Sirt3. Perfusion.

