# Supplementary figures and images for: Exosomes regulate SIRT3-related autophagy by delivering miR-421 to regulate macrophage polarization and participate in OSA-related NAFLD
Source: J Transl Med. 2024 May 19;22:475. doi: 10.1186/s12967-024-05283-8 (PMC11103849; doi:10.1186/s12967-024-05283-8)

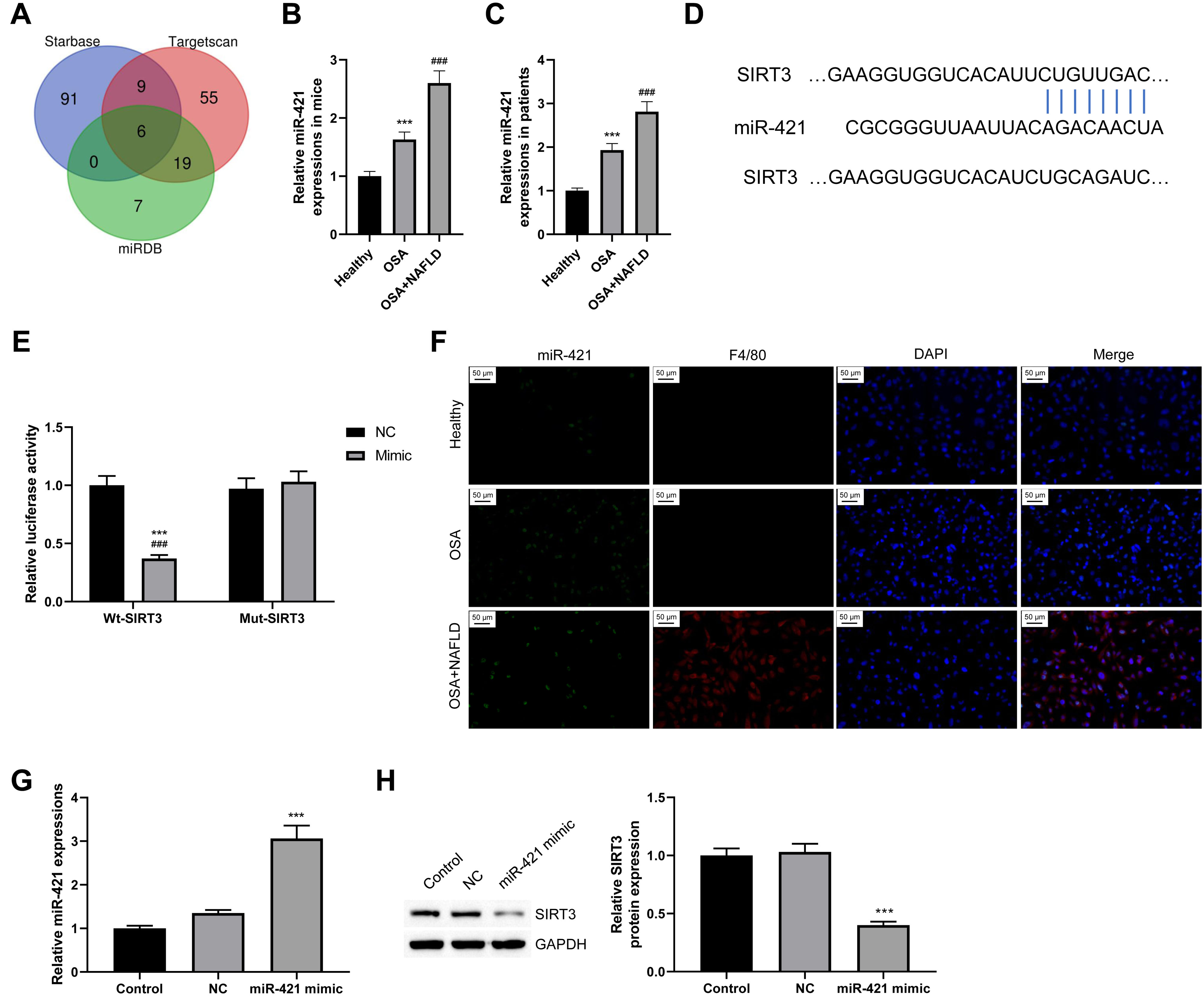

Supplement: Supplementary file 1 — Supplementary Material 1 [file 12967_2024_5283_MOESM1_ESM.jpg]

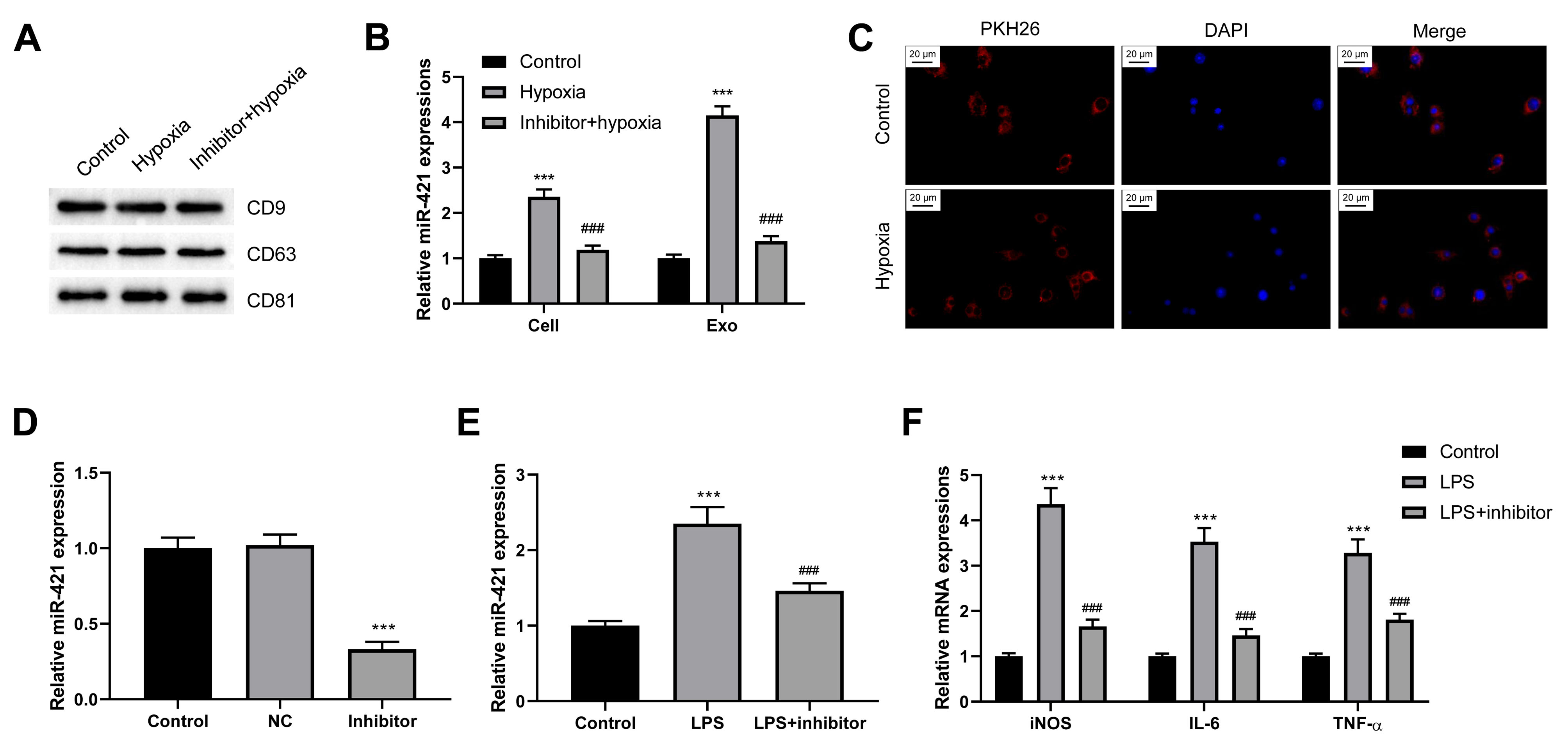

Supplement: Supplementary file 2 — Supplementary Material 2 [file 12967_2024_5283_MOESM2_ESM.jpg]
